# Cognitive and Physical Functioning in Young Adults with Stroke: A Comparative Cross-Sectional Study of Anxiety, Depression, and Stress

**DOI:** 10.3390/neurolint18100192

**Published:** 2026-10-13

**Authors:** Dilek Baykal, Ozge Fircasiguzel, Demet Aygun

**Affiliations:** 1 Department of Nursing, Faculty of Health Sciences, Atlas University, 34408 Istanbul, Türkiye; ozgeakerr@gmail.com; 2 Department of Neurology, Faculty of Medicine, Biruni University, 34408 Istanbul, Türkiye; demetaygun@yahoo.com

**Keywords:** stroke, young adults, stress, anxiety, depression, mental health

## Abstract

**Background:** Stroke in young adults is an increasingly important public health concern because of its long-term impact on cognitive functioning, psychological well-being, and social participation. Understanding the interplay between cognitive impairment, psychological distress, and stroke severity is essential for optimizing post-stroke management. **Objective:** To compare cognitive function and symptoms of depression, anxiety, and stress between young adults with stroke and healthy controls and to investigate the associations of these outcomes with sociodemographic and disease-related characteristics. **Methods:** This cross-sectional case–control study was conducted at a university hospital in Türkiye between January and December 2025. A total of 160 participants, including 80 patients aged 18–<65 years with a clinical diagnosis of stroke and 80 healthy controls, were enrolled. Cognitive function was evaluated using the Mini-Mental State Examination (MMSE), neurological severity using the National Institutes of Health Stroke Scale (NIHSS), functional disability using the Modified Rankin Scale (mRS), and psychological symptoms using the Depression Anxiety Stress Scale-21 (DASS-21). Group differences were examined using multivariate general linear models, and Pearson correlation analyses were performed to evaluate associations between disease-specific characteristics and psychological and cognitive outcomes. **Results:** Across the study groups, the control group exhibited significantly higher levels of depression, anxiety, and stress than the stroke group. These between-group differences remained significant after adjustment for age, whereas the age-adjusted between-group difference in MMSE scores was not statistically significant. Age was not significantly associated with depression, anxiety, or stress scores but was significantly associated with MMSE performance. After adjustment for study group and age, the examined sociodemographic characteristics—gender, education level, employment status, income level, marital status, alcohol use, and smoking—showed no statistically significant main effects on depression, anxiety, stress, or MMSE scores. Although employment status showed a trend toward an association with anxiety, this finding did not reach statistical significance (*p* = 0.053). Higher NIHSS and mRS scores were significantly associated with higher depression, anxiety, and stress scores and lower MMSE scores (all *p* < 0.001). **Conclusions:** Psychological distress and cognitive performance demonstrated distinct patterns of association in young adults following stroke. Neurological severity and functional disability were consistently associated with both psychological distress and cognitive performance. After adjustment for study group and age, the examined sociodemographic characteristics showed no statistically significant main effects on depression, anxiety, stress, or MMSE scores. In contrast, age was significantly associated with MMSE performance but not with depression, anxiety, or stress. The unexpectedly higher levels of depression, anxiety, and stress observed among controls persisted after adjustment for age and warrant further investigation, particularly in relation to potential contextual, psychosocial, and recruitment-related factors. These findings highlight the importance of considering psychological, cognitive, neurological, and functional measures together when assessing young adults with stroke.

## 1. Introduction

Stroke remains a major global cause of death and disability. According to the Global Burden of Disease Study 2021, approximately 11.9 million incident strokes, 93.8 million prevalent strokes, 7.3 million stroke-related deaths, and 160.5 million disability-adjusted life-years (DALYs) were estimated worldwide in 2021. Stroke was the third leading cause of death and the fourth leading cause of DALYs globally in 2021 [1]. Although stroke incidence has generally declined in older populations in recent decades, this decline has not been consistently observed among younger adults. Recent evidence indicates that stroke incidence among individuals younger than 55 years has remained stable or increased in several populations since the beginning of the 21st century, highlighting the growing public health importance of stroke in younger adults [2]. The World Stroke Organization further estimated that the global economic cost of stroke exceeds US$890 billion annually, corresponding to approximately 0.66% of global gross domestic product, underscoring the substantial societal and economic burden of stroke [3]. Cerebrovascular disease is also an important contributor to cognitive impairment and dementia, with vascular cognitive impairment and dementia [4].

The definition of young-onset stroke varies across the literature, and no universally accepted upper age limit has been established. Recent studies have commonly defined young adult stroke using an upper age limit of approximately 45–50 years, with contemporary literature frequently referring to individuals aged 18–49 years as young adults with stroke [5,6]. Other studies have used broader age ranges; for example, a recent study of first-ever ischemic stroke in young adults included individuals aged 18–55 years [7]. Data-driven studies have also suggested cut-off values around 52–54 years, further demonstrating the lack of a universally accepted definition [8,9]. Therefore, definitions of young stroke vary substantially across studies, and findings should be interpreted in the context of the specific age range used in each study.

Beyond its physical consequences, stroke frequently results in cognitive impairment. Post-stroke cognitive impairment (PSCI) affects approximately 39–47% of stroke survivors and commonly involves deficits in attention, memory, executive functioning, and processing speed [10]. Vascular cognitive impairment and dementia (VCID) represent the second most common cause of dementia after Alzheimer’s disease and account for approximately 15–20% of dementia cases worldwide [11]. Recent evidence suggests that cognitive decline is not limited to major strokes. A large cohort study demonstrated that individuals with first-ever stroke exhibited the greatest cognitive decline, while patients with transient ischemic attack (TIA) also experienced measurable deterioration compared with asymptomatic community controls, indicating that even transient cerebrovascular events may have lasting cognitive consequences [12].

Psychological disorders are also common after stroke. Post-stroke depression (PSD) affects approximately one-third of stroke survivors and is associated with poorer functional recovery, reduced quality of life, and increased mortality [13,14]. Post-stroke anxiety is a common neuropsychiatric complication, affecting approximately one-quarter to one-third of stroke survivors. It has been associated with female sex, cognitive impairment, greater functional disability, impaired activities of daily living, and poorer quality of life [15]. Young stroke survivors appear to experience an even greater psychological burden. A recent meta-analysis reported pooled prevalence estimates of 31% for depression and 39% for anxiety among young adults after stroke, while 25% experienced both conditions simultaneously [16].

Cognitive impairment and psychological distress are increasingly recognized as interconnected rather than independent consequences of stroke. Depressive symptoms have been associated with poorer memory performance even among stroke survivors with relatively preserved functional status, and psychiatric symptoms appear to be more strongly associated with subjective cognitive difficulties than with objectively measured cognitive performance [17]. These findings suggest that comprehensive post-stroke assessment should include both objective cognitive evaluation and screening for depression and anxiety to facilitate early identification of patients requiring multidisciplinary rehabilitation.

Despite increasing awareness of these sequelae, most previous studies have focused on older stroke populations, whereas evidence regarding young adults remains limited. Because young stroke survivors face unique educational, occupational, and social challenges, understanding the relationships among cognitive impairment, functional disability, and psychological distress is essential for developing effective rehabilitation strategies tailored to this population.

Psychological distress is an important concern among young adults after stroke, but its occurrence and severity may vary substantially across individuals. A systematic review and meta-analysis published in 2024 reported pooled prevalences of 31% for depressive symptoms and 39% for anxiety symptoms among young adults with stroke, while also demonstrating substantial heterogeneity across studies [16,17]. More recent evidence from a 2026 meta-analysis similarly found that anxiety and depression were common among young stroke patients, with pooled prevalence estimates of approximately 35% for both outcomes. Importantly, neurological severity, lesion-related characteristics, vascular comorbidities, and psychosocial factors were identified as potential factors associated with depression in young stroke populations [18]. These findings suggest that psychological distress after stroke should not be interpreted solely as a direct consequence of having experienced a stroke, but rather as an outcome that may reflect the interaction of neurological impairment, functional consequences, and broader psychosocial circumstances [18]. In particular, individuals with relatively mild stroke and preserved functional independence may experience a different psychological burden from those with more severe disability, while individuals recruited from hospital-based comparison groups may themselves be exposed to family, caregiving, occupational, or financial stressors. Therefore, psychological distress may not necessarily be greater among stroke survivors than among all hospital-based comparison populations, and comparisons between groups should take their clinical and psychosocial context into account.

Therefore, the present study aimed to compare cognitive function and psychological distress between young adults with stroke and healthy controls and to examine the associations between cognitive function, stroke severity, functional disability, and levels of anxiety, depression, and stress among young adults with stroke. The findings highlight specific psychological and physical vulnerabilities in young stroke survivors that warrant targeted, multi-disciplinary support, potentially led by specialized nursing staff.

## 2. Materials and Methods

### 2.1. Study Population

This study is a cross-sectional, descriptive case–control study. The study was conducted between January and December 2025 at a university hospital in Istanbul, including neurology outpatient clinics and internal medicine inpatient units. The study population consisted of young adults diagnosed with stroke and healthy controls recruited from the same hospital environment. An a priori sample size calculation was performed using G*Power version 3.1.9.4. The primary outcome was global cognitive functioning assessed using the Mini-Mental State Examination (MMSE). For the comparison of MMSE scores between the stroke and control groups, a point-biserial correlation model was specified with a two-tailed significance level of α = 0.05, an expected effect size of ρ = 0.30, and 95% statistical power. The analysis indicated a minimum required total sample size of 134 participants. The final study sample comprised 160 participants, including 80 patients with stroke and 80 healthy controls, thus exceeding the calculated minimum sample size. No participants were excluded after enrollment, and no dropout occurred; therefore, all 160 enrolled participants were included in the final analysis.

There is no universally accepted upper age limit for defining young-onset stroke, and published studies have used different age thresholds, most commonly ranging from approximately 45 to 55 years. Recent literature has frequently used 18–49 years to define young adult stroke, whereas other reviews have used an upper limit of 55 years. Therefore, the age range used in the present study (18–<65 years) should be considered a study-specific operational definition rather than a universally accepted definition of young stroke. The upper age limit of 65 years was selected to capture adults experiencing stroke before the conventional older-stroke population while maintaining a clinically feasible sample size. Accordingly, the findings should be interpreted as applying to adults aged 18–65 years with stroke and should not be generalized to younger stroke populations defined by narrower age criteria.

#### Inclusion and Exclusion Criteria

Case Group: Inclusion criteria were age 18–<65 years, confirmed diagnosis of stroke, literacy, and voluntary participation.

Exclusion criteria included a history of psychiatric disorders, current use of psychiatric medications, communication impairments, and the presence of neurological disorders other than stroke.

Control Group: Inclusion criteria were age 18–<65 years, literacy, and voluntary participation.

Exclusion criteria were the presence of any chronic disease or communication difficulties.

### 2.2. Data Collection Instruments

A structured sociodemographic information form was used to collect data on participants’ age, gender, educational level, marital status, and other relevant background characteristics. This form was designed to describe the demographic profile of the study population.

The National Institutes of Health Stroke Scale (NIHSS) was employed to assess the severity of neurological deficits in patients with stroke and to provide prognostic information. The scale consists of 11 items evaluating level of consciousness, response to commands, extraocular movements, visual fields, motor function of the upper and lower extremities, limb ataxia, sensory loss, aphasia, dysarthria, and hemispatial neglect. Total scores range from 0 to 42, with lower scores indicating less severe neurological impairment and better clinical status [19,20].

The Modified Rankin Scale (mRS) was used to evaluate functional dependence and recovery. The scale comprises six categories reflecting the degree of disability and the level of assistance required in daily life. According to the mRS classification, scores of 1–2 indicate functional independence, whereas scores of 3 or higher indicate varying levels of dependency [21].

The Mini-Mental State Examination (MMSE) was administered as a brief and practical screening tool for cognitive impairment. The MMSE is scored out of 30 points, with scores below 24 commonly considered indicative of possible cognitive dysfunction. The instrument assesses multiple cognitive domains, including orientation, attention, memory, language, and motor skills, and can be completed within approximately 10–15 min using simple questions and commands. While the MMSE is widely used for its ease of administration and applicability across diverse patient populations, results should be interpreted with caution in individuals with low educational attainment, as this may affect performance [22].

The Mini-Mental State Examination (MMSE) was administered as a brief and feasible global cognitive screening instrument. The MMSE is scored out of 30 points, with scores below 24 commonly considered indicative of possible cognitive dysfunction. It assesses several cognitive domains, including orientation, attention, memory, language, and motor skills, and can be completed within approximately 10–15 min using simple questions and commands [22]. The MMSE was selected because the present study was designed as a brief, face-to-face clinical assessment and aimed to provide a standardized measure of global cognitive screening performance rather than a comprehensive assessment of post-stroke neurocognitive functioning. However, particularly in relatively young and/or highly educated adults with stroke, clinically relevant difficulties in executive functioning, processing speed, attention, working memory, and other domain-specific abilities may not be adequately captured by the MMSE. Therefore, more comprehensive instruments such as the Montreal Cognitive Assessment (MoCA) or multidomain neuropsychological batteries may be more sensitive to subtle post-stroke cognitive deficits. Results should also be interpreted with caution in individuals with low educational attainment, as educational level may affect MMSE performance. Accordingly, MMSE findings in the present study should be interpreted as measures of global cognitive screening performance rather than evidence that all cognitive domains were preserved.

Psychological symptoms were assessed using the Depression Anxiety Stress Scale–21 (DASS-21), a short form derived from the original 42-item scale and developed by Henry and Crawford (2005) [23]. The Turkish version of the scale was validated by Yılmaz et al. (2017). The DASS-21 consists of 21 items, with seven items each measuring depression, anxiety, and stress. Items are rated on a 4-point Likert scale. In the Turkish adaptation, Cronbach’s alpha coefficients demonstrated good internal consistency for the subscales: 0.808 for anxiety, 0.819 for depression, and 0.755 for stress [24].

### 2.3. Data Collection Procedure

Data were collected through face-to-face interviews by trained researchers after obtaining written informed consent. The raters administering all scales were not blinded to the participants’ group status. Data from participants with stroke were collected from patients hospitalized in the neurology clinic of the university hospital. Control participants were recruited from relatives accompanying patients attending the outpatient clinics of the same university hospital. Recruitment was conducted during routine outpatient visits between January and December 2025. Controls participated voluntarily and completed the sociodemographic information form, the MMSE, and the DASS-21 during face-to-face interviews conducted by trained researchers. No additional assessment of caregiving burden, health-related anxiety, or reasons for accompanying the outpatient was performed.

The MMSE was selected as a brief global cognitive screening instrument to provide a standardized and feasible assessment of overall cognitive status in the study population. However, the MMSE primarily provides a global estimate of cognitive functioning and does not comprehensively assess specific cognitive domains such as executive functioning and processing speed. More extensive domain-specific neuropsychological assessments were not included because the present study was designed as a brief cross-sectional clinical assessment and focused on global cognitive screening rather than comprehensive neuropsychological profiling. Therefore, the MMSE findings should be interpreted as an indicator of global cognitive screening performance rather than as evidence that domain-specific cognitive functions were unaffected.

### 2.4. Statistical Analysis

Statistical analyses were performed using IBM SPSS Statistics version 26.0. Data normality was assessed using the Kolmogorov–Smirnov test together with skewness and kurtosis values. Descriptive statistics were used to summarize participants’ demographic and clinical characteristics. Categorical variables were compared using the chi-square test, while continuous variables were analysed using independent-samples *t* tests. The effects of study group and sociodemographic characteristics on depression, anxiety, stress, and cognitive function were examined using multivariate General Linear Models (GLMs), with age included as a covariate. Given the substantial age imbalance between the stroke and control groups, additional age-adjusted sensitivity analyses were performed for depression, anxiety, stress, and MMSE. Generalized linear models were fitted separately for each outcome, with study group as the fixed factor and age as a continuous covariate. Because age was modeled as a continuous covariate, the age effect was tested with 1 numerator degree of freedom. A normal distribution with an identity link was specified. Because variance heterogeneity was observed for some outcomes, robust covariance estimates were used in these sensitivity analyses. Type III Wald chi-square tests were used to evaluate the effects of study group and age.

Pearson correlation analyses were performed to examine the associations between stroke-related clinical characteristics (NIHSS and mRS scores) and DASS-21 and MMSE scores in the stroke group. All statistical tests were two-tailed, and statistical significance was set at *p* < 0.05. To further assess independent associations with psychological outcomes among participants with stroke, additional multivariable linear regression analyses were performed for depression, anxiety, and stress scores. Separate regression models were constructed for each DASS-21 subscale as the dependent variable. Age, NIHSS score, and income–expenditure status were entered as independent variables. Model fit was evaluated using R^2^ and adjusted R^2^, and the overall significance of each model was assessed using the F test. Unstandardized regression coefficients (B), standardized regression coefficients (β), 95% confidence intervals, and *p*-values were reported for individual predictors. Multicollinearity was assessed using tolerance and variance inflation factor (VIF) values. All statistical tests were two-tailed, and statistical significance was set at *p* < 0.05.

## 3. Results

### 3.1. Descriptive and Clinical Characteristics of the Participants

A total of 160 participants were included in the study, comprising 80 patients with stroke and 80 healthy controls. Patients were significantly older than controls (median age: 52.5 vs. 33.5 years, *p* < 0.001). No significant differences were observed between the groups regarding BMI (*p* = 0.111) or MMSE scores (*p* = 0.169). Similarly, gender, educational level, working status, income, marital status, smoking status, and alcohol use did not differ significantly between the two groups (all *p* > 0.05) (Table 1).

Among the patients, the mean time since stroke onset was 5.78 ± 8.27 months. The mean NIHSS and mRS scores were 5.76 ± 4.20 and 2.41 ± 1.03, respectively. Most patients had experienced an ischemic stroke (81.3%), 48.8% had right hemisphere involvement, and 95.0% had one or more comorbidities (Table 2).

### 3.2. Comparison of DASS and MMSE Scores by Sociodemographic Characteristics

Before interpreting the multivariate GLM results, the assumptions of homogeneity of covariance matrices and homogeneity of error variances were assessed. Box’s M test was significant (Box’s M = 131.042, *F*(10,119,349.801) = 12.746, *p* < 0.001), indicating that the covariance matrices differed between the study groups. Therefore, Pillai’s Trace was used as the primary multivariate statistic because of its relative robustness to violations of the covariance homogeneity assumption. The multivariate effect of study group (stroke vs. control) was significant (Pillai’s Trace = 0.674, *F*(4,155) = 79.942, *p* < 0.001, partial η^2^ = 0.674). Levene’s tests showed significant heterogeneity of error variances for MMSE (*F*(1,158) = 27.603, *p* < 0.001), depression (*F*(1,158) = 3.965, *p* = 0.048), and anxiety (*F*(1,158) = 5.371, *p* = 0.022), whereas the assumption was not violated for stress (*F*(1,158) = 2.123, *p* = 0.147).

A Multivariate General Linear Model (Multivariate GLM) analysis was conducted to determine the main effects of socio-demographic characteristics on the participants’ psychological states (Depression, Anxiety, Stress) and cognitive functions (MMSE).

The comparisons of DASS and MMSE scores according to sociodemographic characteristics are presented in Table 3. Main effects were evaluated for depression, anxiety, stress, and MMSE scores.

Regarding age, no significant main effects were observed for depression, anxiety, or stress scores. Specifically, age was not significantly associated with depression scores (*F*(1,157) = 0.002, *p* = 0.962, ηp^2^ = 0.000, 95% CI [−0.090, 0.085]), anxiety scores (*F*(1,157) = 0.000, *p* = 0.984, ηp^2^ = 0.000, 95% CI [−0.073, 0.075]), or stress scores (*F*(1,157) = 1.020, *p* = 0.314, ηp^2^ = 0.006, 95% CI [−0.120, 0.039]). In contrast, age was significantly associated with MMSE scores (*F*(1,157) = 5.921, *p* = 0.016, ηp^2^ = 0.036, 95% CI [−0.219, −0.023]). The negative regression coefficient for age (B = −0.121, SE = 0.050) indicated that MMSE scores decreased with increasing age in the adjusted model.

Regarding gender, no significant main effects were observed for depression, anxiety, stress, or MMSE scores after adjustment for age and study group. The effects were non-significant for depression (*F*(1,156) = 0.000, *p* = 0.990, partial η^2^ = 0.000), anxiety (*F*(1,156) = 0.000, *p* = 0.998, partial η^2^ = 0.000), stress (*F*(1,156) = 0.932, *p* = 0.336, partial η^2^ = 0.006), and MMSE (*F*(1,156) = 0.427, *p* = 0.514, partial η^2^ = 0.003).

For education level, no significant main effects were observed for depression (*F*(2,157) = 1.885, *p* = 0.154, ηp^2^ = 0.024), anxiety (*F*(2,157) = 0.071, *p* = 0.932, ηp^2^ = 0.001), stress (*F*(2,157) = 1.550, *p*= 0.216, ηp^2^ = 0.020) or MMSE scores (*F*(2,157) = 1.056, *p* = 0.350, ηp^2^ = 0.013). The estimated marginal mean for MMSE was (95% CI: 24.308–25.959).

Employment status was not significantly associated with depression, anxiety, stress, or MMSE scores after adjustment for age and study group. Specifically, the main effect of employment status was not significant for depression, *F*(3,156) = 1.375, *p* = 0.253, partial η^2^ = 0.026; anxiety, *F*(3,156) = 2.611, *p* = 0.053, partial η^2^ = 0.048; stress, *F*(3,156) = 1.634, *p* = 0.184, partial η^2^ = 0.031; or MMSE, *F*(3,156) = 0.199, *p* = 0.897, partial η^2^ = 0.004.

Income category was not significantly associated with any of the four outcome measures after adjusting for age and study group. For depression, there was no significant main effect of income, *F*(2,157) = 0.788, *p* = 0.456, ηp^2^ = 0.010. Similarly, income was not significantly associated with anxiety, *F*(2,157) = 1.428, *p* = 0.243, ηp^2^ = 0.018, stress, *F*(2,157) = 0.518, *p* = 0.597, ηp^2^ = 0.007, or MMSE scores, *F*(2,157) = 1.018, *p* = 0.364, ηp^2^ = 0.013. Thus, after controlling for age and study group, income level did not show a significant effect on depression, anxiety, stress, or cognitive performance.

Marital status was not significantly associated with any of the four outcome measures after adjusting for age and study group. There was no significant main effect of marital status on depression, *F*(1,156) = 0.036, *p* = 0.849, ηp^2^ < 0.001, anxiety, *F*(1,156) = 0.060, *p* = 0.806, ηp^2^ < 0.001, stress, *F*(1,156) = 0.342, *p* = 0.560, ηp^2^ = 0.002, or MMSE scores, *F*(1,156) = 0.111, *p* = 0.739, ηp^2^ = 0.001. Thus, marital status was not significantly associated with depression, anxiety, stress, or cognitive performance after controlling for age and study group.

Alcohol use was not significantly associated with any of the four outcome measures after adjustment for study group and age. Specifically, alcohol use was not significantly associated with depression, *F*(1,156) = 0.013, *p* = 0.909, ηp^2^ = 0.000; anxiety, *F*(1,156) = 0.013, *p* = 0.908, ηp^2^ = 0.000; stress, *F*(1,156) = 0.932, *p* = 0.336, ηp^2^ = 0.006; or MMSE scores, *F*(1,156) = 0.145, *p* = 0.704, ηp^2^ = 0.001. Thus, alcohol use showed no significant main effect on depression, anxiety, stress, or cognitive performance.

Finally, smoking status was not significantly associated with depression (*F*(1,157) = 0.024, *p* = 0.878, ηp^2^ = 0.000), anxiety (*F*(1,157) = 1.498, *p* = 0.223, ηp^2^ = 0.009), stress (*F*(1,157) = 1.223, *p* = 0.271, ηp^2^ = 0.008), or MMSE scores (*F*(1,157) = 0.041, *p* = 0.840, ηp^2^ = 0.000) (Table 3).

Given the significant age difference between the stroke and control groups, additional age-adjusted sensitivity analyses were performed using generalized linear models with age entered as a continuous covariate and robust covariance estimates. After adjustment for age, between-group differences in depression, anxiety, and stress remained statistically significant. For depression, the group effect was significant (Wald χ^2^ = 52.696, df = 1, *p* < 0.001), whereas age was not significant (Wald χ^2^ = 0.002, df = 1, *p* = 0.965). Similarly, the group effect remained significant for anxiety (Wald χ^2^ = 87.879, df = 1, *p* < 0.001), while the effect of age was not significant (Wald χ^2^ = 0.000, df = 1, *p* = 0.984). For stress, a significant group effect was also observed (Wald χ^2^ = 33.593, df = 1, *p* < 0.001), whereas age was not significant (Wald χ^2^ = 0.865, df = 1, *p* = 0.352). In contrast, for MMSE, age remained significantly associated with the outcome (Wald χ^2^ = 5.925, df = 1, *p* = 0.015), while the adjusted group effect was not statistically significant (Wald χ^2^ = 1.103, df = 1, *p* = 0.294). Thus, the significant between-group differences in depression, anxiety, and stress persisted after adjustment for age, whereas the MMSE comparison remained non-significant after accounting for age (Table S1).

In the multivariable linear regression analysis of stress scores among participants with stroke, the overall model was statistically significant (*F*(3,76) = 8.896, *p* < 0.001) and explained 26.0% of the variance in stress scores (R^2^ = 0.260; adjusted R^2^ = 0.231). Age was independently associated with stress scores (B = −0.123, SE = 0.051, β = −0.248, *p* = 0.019, 95% CI: −0.226 to −0.021). NIHSS was positively and independently associated with stress (B = 0.497, SE = 0.113, β = 0.466, *p* < 0.001, 95% CI: 0.272 to 0.723). Income was not independently associated with stress scores (B = 1.093, SE = 0.867, β = 0.133, *p* = 0.211, 95% CI: −0.633 to 2.820). No substantial multicollinearity was observed, with VIF values ranging from 1.103 to 1.153 (Table S2).

No significant correlations were observed between stroke type, affected brain region, or comorbidities and depression, anxiety, stress, or MMSE scores (all *p* > 0.05). NIHSS scores were positively correlated with depression (r = 0.553), anxiety (r = 0.511), and stress (r = 0.444), and negatively correlated with MMSE scores (r = −0.571) (all *p* < 0.001). Likewise, mRS scores showed positive correlations with depression (r = 0.481), anxiety (r = 0.430), and stress (r = 0.434), and a negative correlation with MMSE scores (r = −0.440) (all *p* < 0.001) (Table 4).

## 4. Discussion

The present study investigated psychological distress and cognitive status among adults with stroke compared with healthy controls and further examined the associations of sociodemographic and clinical characteristics with these outcomes. Several findings emerged. First, depression, anxiety, and stress scores differed significantly between the study groups, with higher psychological distress observed among controls, and these differences remained significant after adjustment for age. In contrast, MMSE scores did not differ significantly between the groups after age adjustment. Second, within the stroke group, greater neurological severity and functional disability were consistently associated with greater psychological distress and poorer MMSE performance. Higher NIHSS scores were associated with higher depression, anxiety, and stress scores, while both higher NIHSS and mRS scores were associated with lower MMSE scores. Third, the revised analyses did not identify statistically significant main effects of the examined sociodemographic characteristics on depression, anxiety, stress, or MMSE scores. Employment status showed a near-significant association with anxiety, but this finding did not reach the conventional threshold for statistical significance. In contrast, age was significantly associated with MMSE performance in the age-adjusted analysis. Finally, stroke subtype, affected hemisphere, and comorbid conditions were not significantly associated with the psychological or cognitive outcomes examined. Overall, these findings suggest that psychological and cognitive outcomes after stroke may show different patterns of association, with neurological severity and functional disability demonstrating the most consistent relationships across outcomes.

### 4.1. Psychological Distress and the Unexpected Control-Group Finding

The finding that healthy controls reported higher levels of depression, anxiety, and stress than patients with stroke requires careful interpretation. This finding should not be interpreted as evidence that stroke is generally associated with lower psychological distress. Rather, it may reflect the characteristics of the present cohort and the context in which the control participants were recruited. The stroke group consisted of adults aged 18–65 years and generally demonstrated relatively mild-to-moderate neurological impairment, as reflected by the mean NIHSS score of 5.76 ± 4.20, together with comparatively preserved functional status (mean mRS: 2.41 ± 1.03). Such characteristics may facilitate adaptation and preservation of autonomy and social participation.

The recruitment strategy for the control group is also relevant. Controls were recruited from relatives accompanying patients attending outpatient clinics. These individuals may have experienced caregiving responsibilities, concerns about a family member’s health, illness-related anxiety, or other hospital-related stressors. However, these factors were not directly assessed in the present study. Therefore, selection and contextual factors may have contributed to the higher psychological distress observed among controls. Importantly, the between-group differences in depression, anxiety, and stress remained significant after adjustment for age, whereas age itself was not significantly associated with these outcomes in the revised analyses. Thus, the substantial age difference between the groups alone does not appear to explain the observed between-group differences in psychological distress.

Previous studies focusing specifically on young adults with stroke have reported a substantial burden of psychological symptoms. A 2024 systematic review and meta-analysis of 20 studies involving 4748 young adults with stroke aged 18–55 years reported pooled prevalence estimates of 31% for post-stroke depressive symptoms and 39% for post-stroke anxiety symptoms [16]. More recently, a 2026 meta-analysis including 26 studies and 5634 young stroke patients, defined as individuals aged 15–60 years, reported pooled prevalence estimates of 35% for both anxiety and depression [18]. These findings indicate that psychological symptoms are common among young stroke survivors. However, substantial heterogeneity across studies, including differences in age ranges, assessment instruments, stroke characteristics, and assessment timing, limits direct comparisons with the present findings [16,18].

The present study included adults aged 18–65 years and used the DASS-21 to assess depression, anxiety, and stress symptoms. Comparisons with previous prevalence estimates should therefore be made cautiously because of differences in age ranges, measurement instruments, diagnostic thresholds, and assessment timing. Moreover, the higher depression, anxiety, and stress scores observed among controls in the present study differ from the substantial psychological burden generally reported in young stroke populations. This discrepancy may reflect differences in study populations and recruitment settings. Because controls were recruited from relatives accompanying patients attending outpatient clinics, they may have been exposed to illness-related concerns or other contextual stressors, although these factors were not directly assessed. The findings should consequently be interpreted within the specific recruitment context of the study and should not be generalized to young stroke populations as a whole. Differences in clinical characteristics and psychological assessment methods may also contribute to variation between studies.

### 4.2. Neurological Severity, Functional Disability, and Psychological Outcomes

Despite the higher psychological distress observed among controls, neurological severity was consistently associated with psychological outcomes within the stroke group. Higher NIHSS scores were associated with greater depression, anxiety, and stress symptoms. This finding is consistent with previous research showing that more severe neurological deficits are associated with greater emotional burden after stroke [15,25,26].

Several mechanisms may contribute to this association. Greater neurological impairment may affect mobility, communication, independence, social participation, and the ability to resume previous occupational and family roles. Increased dependence on caregivers and reduced perceived autonomy may further contribute to psychological vulnerability. From a psychosocial perspective, functional limitations may therefore represent an important pathway linking neurological impairment with emotional distress.

Functional disability demonstrated a similar pattern. Higher mRS scores were associated with greater depression, anxiety, and stress symptoms. Functional recovery is closely related to autonomy, participation, and engagement in meaningful daily activities; consequently, persistent disability may be accompanied by greater psychological burden. Conversely, depression and anxiety may adversely affect motivation, rehabilitation participation, and adherence, potentially influencing functional recovery. This possibility is consistent with evidence demonstrating associations between post-stroke depression and poorer functional outcomes [27,28]. However, because the present study was cross-sectional, the directionality of these relationships cannot be established.

The revised analyses did not identify statistically significant main effects of the examined sociodemographic characteristics on depression, anxiety, or stress scores after adjustment for study group and age. Specifically, gender, education level, employment status, income level, marital status, alcohol use, and smoking were not significantly associated with these psychological outcomes. Although employment status showed a trend toward an association with anxiety (*p* = 0.053), this result did not reach the conventional threshold for statistical significance. Age was also not significantly associated with depression, anxiety, or stress scores. These findings indicate that statistically significant associations between the examined sociodemographic characteristics and psychological distress were not detected in the present sample. However, the absence of statistically significant findings should not be interpreted as evidence that these factors have no influence on psychological well-being.

### 4.3. Cognitive Performance and Clinical Characteristics

The cognitive findings showed a different pattern from the psychological outcomes. Although there was no significant between-group difference in MMSE scores after adjustment for age, higher NIHSS and mRS scores were associated with lower MMSE scores within the stroke group. In addition, age was significantly associated with MMSE performance in the revised analysis. Thus, global cognitive performance was more clearly related to neurological severity and functional disability than to the sociodemographic characteristics examined.

The absence of a significant difference in MMSE scores between patients with stroke and controls should nevertheless be interpreted cautiously. The MMSE is a useful measure of global cognitive status but has limited sensitivity for subtle deficits in executive functioning, processing speed, attention, memory, and other domain-specific abilities. Recent lesion-network mapping research has demonstrated that post-stroke cognitive impairment is associated with network-level disconnection patterns, and that attention and processing speed may be particularly sensitive domains of cognitive dysfunction [29]. This limitation may be particularly relevant in younger adults with stroke, who may maintain relatively preserved orientation and basic cognitive abilities while experiencing more subtle deficits. Moreover, age was significantly associated with MMSE scores in the revised analysis, although the age-adjusted between-group effect remained non-significant. Therefore, the absence of a significant group difference does not necessarily indicate complete preservation of cognitive functioning.

The lack of significant associations between MMSE scores and gender, education, employment, income, marital status, alcohol use, or smoking should similarly be interpreted as an absence of statistically detectable associations in this sample rather than evidence that these factors have no influence on cognition. Education and socioeconomic circumstances, for example, may contribute to cognitive reserve, but such effects may not be adequately captured by a brief global screening instrument. The present results therefore suggest that age, neurological severity and functional disability were more clearly associated with MMSE performance than the sociodemographic variables examined.

### 4.4. Stroke Subtype, Lesion Characteristics, and Comorbidities

Stroke subtype, affected hemisphere, and comorbid conditions were not significantly associated with the psychological or cognitive outcomes examined. This finding does not necessarily indicate that lesion characteristics or comorbidities are irrelevant to post-stroke outcomes. Lesion location may influence specific cognitive or emotional domains, while its relationship with global cognitive functioning is likely to depend on lesion burden, network disruption, pre-existing vascular pathology, and overall neurological severity [22,30,31].

Recent evidence suggests that spatially heterogeneous lesions may converge on common patterns of network disruption, meaning that functional consequences may not be explained adequately by anatomical location alone [22]. Similarly, the effects of comorbidities may be difficult to detect in relatively homogeneous populations with a high prevalence of vascular risk factors. In this context, the present findings suggest that global indicators of neurological severity and functional disability may provide clinically useful information when evaluating psychological and cognitive outcomes, although they should not be considered substitutes for detailed neurological or neuroimaging assessment.

The association between neurological severity and psychological distress may have both biological and psychosocial explanations. From a biological perspective, greater neurological injury may involve disruption of neural systems involved in emotional regulation and cognitive processing. Previous literature has implicated neuroinflammation, altered immune signaling, neurotransmitter dysregulation, hypothalamic–pituitary–adrenal axis abnormalities, impaired neuroplasticity, and disruption of functional brain networks in post-stroke psychological disturbances [32,33,34]. These mechanisms provide a plausible biological context for the present findings, particularly the association between greater neurological severity and greater psychological distress. However, these mechanisms were not directly assessed in this study and therefore should be regarded as potential explanations rather than mechanisms demonstrated by the present data.

From a psychosocial perspective, neurological impairment may influence emotional well-being through reduced independence, impaired mobility, limitations in activities of daily living, reduced social participation, and difficulty resuming previous occupational and family roles. Greater disability may increase caregiver dependence and reduce perceived self-efficacy, potentially increasing psychological burden. The 2024 systematic review by Butsing et al. reported associations between post-stroke depression and poorer functional outcomes across multiple domains [14]. A broader biopsychosocial perspective similarly emphasizes the interaction between physical disability, social participation, stress, and social support in shaping post-stroke emotional outcomes [14,35].

A similar multifactorial interpretation may apply to cognitive performance. Greater neurological injury may increase the likelihood of disruption to neural networks supporting attention, executive functioning, processing speed, memory, and other cognitive processes. Functional disability and reduced participation may also influence opportunities for cognitive engagement and recovery. The association between NIHSS and mRS scores and lower MMSE performance in the present study is therefore compatible with a multifactorial model of post-stroke cognition. Nevertheless, because the present study did not include detailed neuropsychological testing or biological markers, these mechanisms remain interpretative rather than directly demonstrated.

Taken together, the present findings suggest that psychological distress and cognitive performance after stroke should not be considered manifestations of a single underlying process. Higher neurological severity and functional disability were associated with greater psychological distress and poorer MMSE performance. In contrast, the revised analyses did not identify statistically significant main effects of the examined sociodemographic characteristics on depression, anxiety, or stress after adjustment for study group and age. Age was significantly associated with MMSE performance, whereas the other sociodemographic characteristics examined did not show statistically significant main effects on MMSE scores.

The unexpectedly higher psychological distress observed among controls should be interpreted within the specific recruitment and demographic context of the study and should not be generalized to stroke populations as a whole. Similarly, the absence of a significant between-group difference in MMSE should not be interpreted as evidence that stroke has no cognitive consequences, particularly given the significant associations of age, NIHSS, and mRS with MMSE performance.

Clinically, these findings support the value of assessing psychological symptoms and cognitive function alongside conventional neurological and functional measures. Patients presenting with greater neurological severity or functional disability may warrant particular attention during psychological and cognitive screening. Future prospective, multicenter studies should incorporate more sensitive cognitive measures such as the MoCA or comprehensive neuropsychological batteries, together with detailed assessments of rehabilitation exposure, medication use, social support, caregiving burden, and socioeconomic circumstances. Longitudinal designs would also help clarify the temporal and potentially bidirectional relationships between neurological impairment, functional recovery, psychological distress, and cognitive performance.

### 4.5. Limitations of the Study

Several limitations should be acknowledged when interpreting the findings of the present study. First, the cross-sectional design does not allow causal or temporal relationships to be established between stroke-related characteristics, psychological distress, and cognitive performance. Although higher NIHSS and mRS scores were associated with greater psychological distress and poorer MMSE performance, these associations should not be interpreted as evidence of causality or directionality. In addition, potentially important confounding factors, including post-stroke rehabilitation, medication use, and social support, were not systematically assessed or controlled for. These factors may influence both psychological and cognitive outcomes, and their potential effects cannot be excluded.

Second, the study was conducted at a single university hospital, which may limit the generalizability of the findings to other clinical settings and to broader populations of young adults with stroke. The relatively modest sample size may also have limited statistical power to detect smaller associations, particularly in subgroup analyses. Future multicenter studies with larger and more representative samples are therefore warranted.

Third, the control-group recruitment strategy represents an important limitation. Healthy controls were recruited through convenience sampling from relatives accompanying patients attending the same hospital. Consequently, the control group may not adequately represent the general healthy population and may have been exposed to caregiving responsibilities, concerns regarding a family member’s health, hospital-related stress, or other contextual factors. These factors were not systematically assessed and may have contributed to the higher depression, anxiety, and stress scores observed among controls. Similarly, broader occupational, socioeconomic, and societal stressors during the recruitment period were not comprehensively measured. Therefore, the possibility of selection or contextual bias cannot be excluded, and the unexpected psychological differences between the groups should be interpreted cautiously.

Fourth, psychological symptoms and cognitive function were assessed using standardized screening instruments rather than clinical diagnostic or comprehensive neuropsychological evaluations. Accordingly, the DASS-21 findings represent symptom severity rather than formal psychiatric diagnoses. Likewise, the MMSE provides an estimate of global cognitive status but may have limited sensitivity to subtle deficits in executive functioning, processing speed, attention, working memory, and other domain-specific abilities. This limitation may be particularly relevant in younger adults with relatively preserved global cognitive functioning. The relatively high MMSE scores observed in both groups also raise the possibility of a ceiling effect, which may have reduced the ability of the instrument to detect small between-group differences.

Importantly, the absence of a significant between-group difference in MMSE scores should therefore not be interpreted as evidence of intact cognitive functioning across all domains. Similarly, the absence of statistically significant associations between sociodemographic characteristics and MMSE performance should not be interpreted as evidence that these factors have no influence on cognition; rather, such associations were not detected using the measure and sample employed in the present study. The significant associations observed between NIHSS, mRS, and MMSE further indicate that global cognitive performance was related to neurological severity and functional disability despite the absence of a significant age-adjusted between-group difference.

Finally, the study did not comprehensively assess several potentially relevant psychosocial and clinical factors, including caregiving burden, social support, rehabilitation exposure, medication use, occupational circumstances, and socioeconomic stressors. Their potential contribution to psychological and cognitive outcomes therefore remains uncertain. Future prospective, multicenter studies should incorporate these variables into longitudinal and multivariable analyses and should use more sensitive cognitive measures, such as the Montreal Cognitive Assessment, together with comprehensive neuropsychological assessment when feasible. Such approaches would help clarify subtle cognitive differences and better characterize the temporal and potentially bidirectional relationships among neurological severity, functional disability, psychological distress, and cognitive functioning after stroke.

## 5. Conclusions

This study demonstrates that psychological distress and cognitive status after stroke show distinct patterns of association with clinical and sociodemographic characteristics. Psychological outcomes were associated with selected sociodemographic characteristics, but these associations were domain-specific rather than uniform across depression, anxiety, and stress. In contrast, none of the examined sociodemographic characteristics showed a significant main effect on MMSE performance. Higher NIHSS and mRS scores were consistently associated with greater psychological distress and lower MMSE scores, indicating that neurological severity and functional disability were the most consistent clinical correlates of both psychological and global cognitive outcomes in this cohort.

Although the stroke group demonstrated lower depression, anxiety, and stress scores than the control group, this finding should be interpreted cautiously. The difference persisted after adjustment for age but may have been influenced by the characteristics and recruitment context of the control group. The relatively mild-to-moderate neurological impairment and comparatively preserved functional status of the stroke participants may also have contributed to the observed psychological profile. Therefore, the present findings should not be interpreted as evidence that stroke is generally associated with lower psychological distress.

The absence of a significant age-adjusted between-group difference in MMSE scores, together with the lack of significant associations between sociodemographic characteristics and MMSE performance, should also be interpreted cautiously. These findings do not establish the absence of cognitive difficulties or demonstrate that sociodemographic factors are unrelated to cognition. Rather, the significant associations of NIHSS and mRS with MMSE performance suggest that neurological severity and functional disability were more clearly related to global cognitive screening performance in this cohort. Given the limited sensitivity of the MMSE to subtle, domain-specific cognitive deficits, more comprehensive assessment may identify cognitive difficulties that were not detected in the present analysis.

Overall, these findings support the importance of considering psychological symptoms, cognitive functioning, neurological severity, and functional disability together in the assessment of adults with stroke. Systematic psychological and cognitive screening may be particularly relevant for patients with greater neurological impairment or functional disability. Future larger, prospective, multicenter studies using multidomain cognitive assessment and more comprehensive evaluation of psychosocial, rehabilitation, and clinical factors are needed to clarify the temporal and clinical significance of these associations and to determine their implications for long-term recovery and quality of life.

## Figures and Tables

**Table 1:** Comparison of participants’ descriptive characteristics.

		**Patients (*n* = 80)**	**Control (*n* = 80)**	***t***	***p***
Age (Mean ± SD)		(51.57 ± 9.03)	(32.31 ± 6.85)	15.195	<0.001
BMI (Mean ± SD)		(28.36 ± 4.40)	(27.16 ± 5.06)	1.603	0.111
MMSE (Mean ± SD)		(24.68 ±6.43)	(25.80 ± 3.22)	−1.382	0.169
		*n* (%)	*n* (%)	χ^2^	*p*
Gender	Male	47 (58.7)	44 (55)	3.025	0.082
	Female	33 (41.3)	36 (45)		
Education	Primary school	15 (18.8)	22 (27.5)	17.236	0.051
	High school	15 (18.8)	30 (37.5)		
	University	50 (62.4)	28 (35)		
Employment status	Working	36 (45)	19 (23.8)	9.711	0.374
	Retired	22 (27.5)	18 (22.5)		
	Housewives	22 (27.5)	22 (27.5)		
	Others	0 (0)	21 (26.2)		
Income	Income > Expense	18 (22.5)	36 (45)	9.059	0.060
	Income = Expense	55 (68.7)	14 (17.5)		
	Income < Expense	7 (8.8)	30 (37.5)		
Marital status	Single	4 (5)	27 (33.7)	0.512	0.774
	Married	76 (95)	53 (66.3)		
Smoking	Yes	28 (35)	43 (53.8)	3.625	0.062
	No	52 (65)	37 (46.2)		
Alcohol use	Yes	3 (3.7)	43 (53.8)	0.157	0.524
	No	77 (96.3)	37 (46.2)		

*t*: Independent samples *t* test, χ^2^: Chi-square test.

**Table 2:** Disease-specific characteristics of the study participants.

Duration of stroke (Min–Max) (M ± SD) (Months)		(3–49) (5.78 ± 8.27)
NIHSS (Min–Max) (M ± SD)		(0–22) (5.76 ± 4.20)
mRS (Min–Max) (M ± SD)		(0–5) (2.41 ± 1.03)
		*n* (%)
Type of stroke	Ischemic	65 (81.3)
	Hemorrhagic	15 (18.7)
Affected region	Right hemisphere	39 (48.8)
	Left hemisphere	41 (51.2)
Comorbidities	Yes	76 (95)
	No	4 (5)

**Table 3:** Comparison of DASS and MMSE Scores by Sociodemographic Characteristics.

Category		Depression		Anxiety		Stress		MMSE	
		Patient	Control	Patient	Control	Patient	Control	Patient	Control
Age		5.78 ± 4.85	14.4 ± 3.99	4.86 ± 4.20	13.83 ± 0.42	6.85 ± 4.48	14.36 ± 3.56	24.68 ± 6.43	25.80 ± 3.22
	Main effect	*F*(1,157) = 0.002 η^2^p = 0.000 *p* = 0.962 95% CI [−0.090, 0.085]		*F*(1,157) = 0.000 η^2^p = 0.000 *p* = 0.984 95% CI [−0.073, 0.075]		*F*(1,157) = 1.020 η^2^p = 0.006 *p* = 0.314 95% CI [−0.120, 0.039]		*F*(1,157) = 5.921 η^2^p = 0.036 *p* = 0.016 95% CI [−0.219–−0.023]	
Gender	Male	5.93 ± 5.09	14.70 ± 4.26	4.65 ± 4.38	14.05 ± 2.96	6.85 ± 4.77	14.13 ± 3.58	24.76 ± 6.57	25.84 ± 3.47
	Female	5.57 ± 4.56	14.02 ± 2.66	5.15 ± 3.98	13.56 ± 3.61	6.84 ± 4.10	14.63 ± 3.57	24.57 ± 6.33	25.75 ± 2.54
	Main effect	*F*(1,156) = 0.000 η^2^p ^=^ 0.000 *p* = 0.990 95% CI [−1.449, 1.429]		*F*(1,156) = 0.00 η^2^p = 0.000 *p* = 0.998 95% CI [−1.216, 1.219]		*F*(1,156) = 0.932 η^2^p = 0.006 *p* = 0.336 95% CI [−0.666, 1.939]		*F*(1,156) = 0.427 η^2^p = 0.003 *p* = 0.514 95% CI [−1.081, 2.149]	
Education	Primary	4.60 ± 2.97	12.63 ± 4.05	4.13 ± 3.18	14.00 ± 3.28	5.73 ± 3.10	15.50 ± 4.36	22.13 ± 8.36	25.63 ± 3.56
	High	5.93 ± 4.14	15.00 ± 2.88	4.66 ± 3.03	14.10 ± 3.69	6.40 ± 4.45	13.66 ± 3.13	24.66 ± 5.86	26.46 ± 2.67
	University	6.10 ± 5.48	15.14 ± 4.63	5.14 ± 4.76	13.39 ± 2.79	7.32 ± 4.82	14.21 ± 3.18	25.46 ± 5.86	25.21 ± 3.46
	Main effect	*F*(2,157) = 1.885 η^2^p = 0.024 *p* = 0.154 95% CI [9.205, 10.666]		*F*(2,157) = 0.071 η^2^p = 0.001 *p* = 0.932 95% CI [8.753, 9.935]		*F*(2,157) = 1.550 η^2^p = 0.020 *p* = 0.216 95% CI [9.755, 11.083]		*F*(2,157) = 1.056 η^2^p = 0.013 *p* = 0.350 95% CI [24.308–25.959]	
Employment status	Employed	4.63 ± 4.46	15.26 ± 4.18	3.77 ± 3.96	13.21 ± 3.29	5.69 ± 4.49	13.21 ± 3.79	25 ± 6.82	26.15 ± 3.74
	Retired	7.40 ± 5.28	14.27 ± 4.75	5.86 ± 4.58	13.62 ± 3.50	8.72 ± 4.22	15.44 ± 2.68	24.86 ± 5.13	25.38 ± 2.27
	Housew.	6.04 ± 4.74	14.59 ± 4.19	5.63 ± 3.95	15.09 ± 2.67	6.86 ± 4.25	13.77 ± 3.36	24 ± 7.15	25.63 ± 3.38
	Others	-	14.61 ± 3.48	-	13.23 ± 3.43	-	15.09 ± 3.98	-	26 ± 3.42
	Main effect	*F*(3,156) = 1.375 η^2^p = 0.026 *p* = 0.253 95% CI [9.523, 11.021]		*F*(3,156) = 2.611 η^2^p = 0.048 *p* = 0.053 95% CI [8.712, 9.965]		*F*(3,156) = 1.634 η^2^p = 0.031 *p* = 0.184 95% CI [9.801, 11.157]		*F*(3,156) = 0.199 η^2^p = 0.004 *p* = 0.897 95% CI [24.441, 26.143]	
Income	Inc. > Exp.	3.55 ± 3.92	13.83 ± 3.75	2.94 ± 2.77	13.91 ± 3.45	5.27 ± 4.40	15.19 ± 3.46	27.11 ± 5.32	25.05 ± 3.25
	Inc. = Exp.	6.18 ± 4.73	15.35 ± 3.38	5.10 ± 4.10	13.07 ± 3.64	7.10 ± 4.28	15.14 ± 4.29	24.14 ± 6.72	25.71 ± 3.02
	Inc. < Exp.	8.42 ± 6.34	14.63 ± 4.52	7.85 ± 6.09	14.06 ± 2.87	8.85 ± 5.61	13 ± 2.95	22.71 ± 5.82	26.73 ± 3.13
	Main effect	*F*(2,157) = 0.788 η^2^p = 0.010 *p* = 0.456 95% CI [9.287, 10.744]		*F*(2,157) = 1.428 η^2^p = 0.018 *p* = 0.243 95% CI [8.814, 10.041]		*F*(2,157) = 0.518 η^2^p = 0.007 *p* = 0.597 95% CI [9.906, 11.230]		*F*(2,157) = 1.018 η^2^p = 0.013 *p* = 0.364 95% CI [24.586–26.220]	
Marital status	Single	5.00 ± 5.83	14.67 ± 3.32	3.75 ± 4.50	14.15 ± 3.34	7.75 ± 3.40	14.12 ± 3.57	27.75 ± 2.87	25.78 ± 2.94
	Married	5.83 ± 4.84	14.26 ± 4.32	4.92 ± 4.21	13.66 ± 3.24	6.80 ± 4.54	14.91 ± 3.54	24.52 ± 6.54	25.83 ± 3.88
	Main effect	*F*(1,156) = 0.036 η^2^p = 0.000 *p* = 0.849 95% CI [9.239, 11.061]		*F*(1,156) = 0.060 η^2^p = 0.000 *p* = 0.806 95% CI [−1.413, 1.814]		*F*(1,156) = 0.342 η^2^p = 0.002 *p* = 0.560 95% CI [9.937, 11.589]		*F*(1,156) = 0.111 η^2^p = 0.001 *p* = 0.739 95% CI [24.110, 26.156]	
Alcohol use	Yes	4.00 ± 4.58	14.58 ± 4.07	3.00 ± 2.64	13.90 ± 3.30	5 ± 4.58	14.02 ± 3.83	28 ± 1.73	25.69 ± 3.58
	No	5.86 ± 4.88	14.18 ± 3.94	4.93 ± 4.24	13.72 ± 3.25	6.92 ± 4.49	14.75 ± 3.22	24.55 ± 6.52	25.91 ± 2.80
	Main effect	*F*(1,156) = 0.013 η^2^p = 0.000 *p* = 0.909 95% CI [9.315, 10.920]		*F*(1,156) = 0.013 η^2^p = 0.000 *p* = 0.908 95% CI [8.645, 10.003]		*F*(1,156) = 0.932 η^2^p = 0.006 *p* = 0.336 95% CI [9.705, 11.158]		*F*(1,156) = 0.145 η^2^p = 0.001 *p* = 0.704 95% CI [24.428, 26.231]	
Smoking	Yes	6.28 ± 5.37	13.97 ± 3.67	4.89 ± 4.63	14.46 ± 3.35	7.46 ± 4.78	14.60 ± 3.42	24.71 ± 7.15	25.88 ± 3.09
	No	5.51 ± 4.58	14.89 ± 4.33	4.84 ± 4.00	13.08 ± 3.02	6.51 ± 4.32	14.08 ± 3.74	24.67 ± 6.08	25.70 ± 3.41
	Main effect	*F*(1,157) = 0.024 η^2^p = 0.000 *p* = 0.878 95% CI [−1.54, 1.32]		*F*(1,157) = 1.498 η^2^p = 0.009 *p* = 0.223 95% CI [8.602, 10.170]		*F*(1,157) = 1.223 η^2^p = 0.008 *p* = 0.271 95% CI [9.743, 11.553]		*F*(1,157) = 0.041 η^2^p = 0.000 *p* = 0.840 95% CI [24.462, 26.044]	

F statistics and partial η^2^ values were obtained from the General Linear Model analyses. Because Box’s M indicated violation of covariance-matrix homogeneity, Pillai’s Trace was used as the primary multivariate test statistic.

**Table 4:** Correlation of DASS-21 and MMSE Scores by Disease-Specific Characteristics.

	Depression	Anxiety	Stress	MMSE
Type of stroke	r = −0.052 *p* = 0.648	r = −0.061 *p* = 0.592	r = −0.049 *p* = 0.669	r = 0.049 *p* = 0.669
Affected region	r = 0.004 *p* = 0.974	r = −0.038 *p* = 0.737	r = −0.095 *p* = 0.404	r = −0.075 *p* = 0.508
Comorbidities	r = −0.037 *p* = 0.742	r = 0.049 *p* = 0.668	r = 0.008 *p* = 0.946	r = −0.034 *p* = 0.767
NIHSS	r = 0.553 *p* < 0.001	r = 0.511 *p* < 0.001	r = 0.444 *p* < 0.001	r = −0.571 *p* < 0.001
mRS	r = 0.481 *p* < 0.001	r = 0.430 *p* < 0.001	r = 0.434 *p* < 0.001	r = −0.440 *p* < 0.001

DASS-21, Depression Anxiety Stress Scale-21; MMSE, Mini-Mental State Examination; NIHSS, National Institutes of Health Stroke Scale; mRS, modified Rankin Scale. All correlations were calculated using Pearson correlation analysis.

## Data Availability

The data presented in this study are available on request from the corresponding author. The data are not publicly available due to privacy restrictions pertaining to patient information.

## Supplementary Materials

The following supporting information can be downloaded at: https://www.mdpi.com/article/10.3390/neurolint18100192/s1, Table S1: Robust age-adjusted sensitivity analyses for cognitive and psychological outcomes, Table S2: Multivariable Linear Regression Analysis of Depression, Anxiety, and Stress Scores Among Participants with Stroke.

## References

[1] GBD 2021 Stroke Risk Factor Collaborators (2024). Global, regional, and national burden of stroke and its risk factors, 1990–2021: A systematic analysis for the Global Burden of Disease Study 2021. Lancet Neurol..

[2] Nehme A., Li L. (2026). The rising incidence of stroke in the young: Epidemiology, causes and global impact. Int. J. Stroke..

[3] Feigin V.L., Brainin M., Norrving B., Martins S.O., Pandian J., Lindsay P.F., Grupper M., Rautalin I. (2025). World Stroke Organization: Global Stroke Fact Sheet 2025. Int. J. Stroke..

[4] Sachdev P.S., Bentvelzen A.C., Gustafson D., Hansra G.K., Hosoki S., Jiang J., Kalaria R. (2026). Vascular Cognitive Impairment and Dementia: Clinical Features, Neuropathology, and Biomarkers. J. Am. Coll. Cardiol..

[5] Rasing A., Hilkens N.A., Leeuw F.E. (2026). Young stroke: An update on epidemiology, emerging risk factors, and future research directions. Int. J. Stroke.

[6] Tan B.Y., Tan S.M.L., Ng M.B.J., Chan B.P. (2026). Ischaemic stroke in young adults: An overview of the classification, risk factors, aetiologies and outcomes. Singap. Med. J..

[7] Schneider T.R., Dittrich T.D., Kahles T., Katan M., Luft A.R., Mono M.L., Bolognese M., Arnold M., Heldner M., Michel P. (2025). First ischemic stroke in young adults: Sex and age-related differences in stroke rates, risk factors, and etiologies. Eur. Stroke J..

[8] Abedi V., Lambert C., Chaudhary D., Rieder E., Avula V., Hwang W., Li J., Zand R. (2023). Defining the Age of Young Ischemic Stroke Using Data-Driven Approaches. J. Clin. Med..

[9] Takeshita S., Ogata T., Arima H., Tsuboi Y. (2021). Proposed definition for young-onset ischemic stroke according to its cause. Clin. Neurol. Neurosurg..

[10] Ma Y., Yang Y., Wang X., Huang Y., Nan J., Feng J., Yan F., Han L. (2025). Prevalence and Risk Factors of Poststroke Cognitive Impairment: A Systematic Review and Meta-Analysis. Public Health Nurs..

[11] Mok V.C.T., Cai Y., Markus H.S. (2024). Vascular cognitive impairment and dementia: Mechanisms, treatment, and future directions. Int. J. Stroke.

[12] Del Bene V.A., Howard G., Gropen T.I., Lyerly M.J., Howard V.J., Sawyer R.P., Lazar R.M. (2025). Cognitive Decline After First-Time Transient Ischemic Attack. JAMA Neurol..

[13] Naghedi A., Delgado-Mederos R., Vives-Bauza C. (2025). Stroke Survivors Have Almost Three Times Higher Risk of Depression: A Systematic Review and Meta-Analysis. J. Clin. Med..

[14] Butsing N., Zauszniewski J.A., Ruksakulpiwat S., Griffin M.T.Q., Niyomyart A. (2024). Association between post-stroke depression and functional outcomes: A systematic review. PLoS ONE.

[15] Ruthmann F., Lo J.W., Mendyk-Bordet A.M., Allart E., Köhler S., Klimkowicz-Mrowiec A., STROKOG Collaboration (2025). Prevalence of poststroke anxiety and its associations with global cognitive impairment: An individual participant data analysis. J. Affect. Disord..

[16] Ignacio K.H.D., Muir R.T., Diestro J.D.B., Singh N., Yu M.H.L.L., Omari O.E., Abdalrahman R., Barker-Collo S.L., Hackett M.L., Dukelow S.P. (2024). Prevalence of depression and anxiety symptoms after stroke in young adults: A systematic review and meta-analysis. J. Stroke Cerebrovasc. Dis..

[17] Kliem E., Gjestad E., Ryum T., Olsen A., Thommessen B., Indredavik B., Bieliauskas L., Due-Tønnessen P., Fladby T., Grambaite R. (2022). The Relationship of Psychiatric Symptoms with Performance-Based and Self-Reported Cognitive Function After Ischemic Stroke. J. Int. Neuropsychol. Soc..

[18] Tong Y., Cao S., Wang J., Qin Y., Lin J., Fang Z., Qiu J. (2026). Prevalence of anxiety and depression in young stroke patients, and associated factors: A meta-analysis. Front. Neurol..

[19] Lyden P.D., Lu M., Levine S.R., Brott T.G., Broderick J. (2001). NINDS rtPA Stroke Study Group. A modified National Institutes of Health Stroke Scale for use in stroke clinical trials: Preliminary reliability and validity. Stroke.

[20] Sümer M., Benbir G., Can U., Kutluk K., Uzuner N. (2015). Scales: Guidelines of Turkish Society of Cerebrovascular Diseases-2015. Turk. J. Cerebrovasc. Dis..

[21] Rundek T., Sacco R.L. (2004). Outcome following stroke. Stroke.

[22] Güngen C., Ertan T., Eker E., Yaşar R., Engin F. (2002). Reliability and Validity of the Standardized Mini Mental State Examination in the Diagnosis of Mild Dementia in Turkish Population. Türk Psikiyatr. Derg..

[23] Henry J.D., Crawford J.R. (2005). The short-form version of the Depression Anxiety Stress Scales (DASS-21): Construct validity and normative data in a large non-clinical sample. Br. J. Clin. Psychol..

[24] Yılmaz Ö., Boz H., Arslan A. (2017). The Validity and Reliability of Depression Stress and Anxiety Scale (DASS 21) Turkish Short Form. Res. Financ. Econ. Soc. Stud..

[25] Zhang S., Yuan Y., Zhuang W., Xiong T., Xu Y., Zhang J., Tao C., Liang J., Wang Y. (2022). Contributing Factors and Induced Outcomes of Psychological Stress Response in Stroke Survivors: A Systematic Review. Front. Neurol..

[26] Nelson S.E., El Husseini N., Bahouth M.N., Fleming T.K., Harvey R.L., Hayman L.L., Council on Quality of Care and Outcomes Research (2026). Social and Environmental Determinants of Stroke Functional Outcome and Strategies to Reduce Inequities: A Scientific Statement from the American Heart Association. Stroke.

[27] Ifejika N.L., Awosika O.O., Black T., Duncan P.W., Harvey R.L., Katz D.I., Reeves M.J. (2025). Improving access to stroke rehabilitation and recovery: A policy statement from the American Heart Association/American Stroke Association. Stroke.

[28] Zrelak P.A., Seagraves K.B., Belagaje S., Dusenbury W., García J.J., Hadidi N.N., American Heart Association Council on Cardiovascular and Stroke Nursing and Council on Lifestyle and Cardiometabolic Health (2024). Nursing’s role in psychosocial health management after a stroke event: A scientific statement from the American Heart Association. Stroke.

[29] Zhou Y., Wang Y., Jing J., Li J., Liu H., Wang Y., Liu T., Li S. (2026). Lesion network mapping for post-stroke cognitive impairment. Int. J. Stroke.

[30] Schellekens M.M., Li H., Wijnands M., Papounidou A., Boot E.M., Verhoeven J.I., Tuladhar A.M. (2025). Lesion locations are associated with cognitive impairment after ischemic stroke in young adults. NeuroImage Clin..

[31] Pantoni L., Pikija S., Nguyen T.V., Brainin M. (2026). Recognizing, managing, and preventing cognitive sequelae after stroke. A narrative review. Cereb. Circ. Cogn. Behav..

[32] Zhou H., Wei Y.J., Xie G.Y. (2024). Research progress on post-stroke depression. Exp. Neurol..

[33] Xiao M., Chen Y., Mu J. (2024). Innate immunity-mediated neuroinflammation promotes the onset and progression of post-stroke depression. Exp. Neurol..

[34] Gao L., Song J., Zhao M., Wang R., Chen Y., Li M., An H., Zhou Z., Tan W., Huang Z. (2026). Research advances on the pathogenesis and clinical interventions of post-stroke depression. Front. Neurol..

[35] Lin M.C., Huang S.S. (2025). Diagnosis and etiology of poststroke depression: A review. World J. Psychiatry.

