# Supplementary material for: Cognitive and Physical Functioning in Young Adults with Stroke: A Comparative Cross-Sectional Study of Anxiety, Depression, and Stress
Source: Neurol Int. 2026 Oct 13;18(10):192. doi: 10.3390/neurolint18100192 (PMC13708236; doi:10.3390/neurolint18100192)
Supplement: Supplementary file 1 [file neurolint-18-00192-s001.zip › neurolint-4546385-supplementary.pdf]

Table S1. Robust age-adjusted sensitivity analyses for cognitive and psychological outcomes

Outcomes	Effect	Wald χ^2	df	p	B	95% Wald CI
Depression	Group	52.696	1	<0.001	-8.572	-10.887 to -6.258
	Age	0.002	1	0.965	-0.002	-0.096 to 0.092
Anxiety	Group	87.879	1	<0.001	-8.977	-10.854 to -7.100
	Age	0.000	1	0.984	0.001	-0.071 to 0.073
Stress	Group	33.593	1	<0.001	-6.731	-9.007 to -4.455
	Age	0.865	1	0.352	-0.041	-0.126 to 0.045
MMSE	Group	1.103	1	0.294	1.220	-1.057 to 3.496
	Age	5.925	1	0.015	-0.121	-0.219 to -0.024

Generalized linear models included group as the fixed factor and age as a continuous covariate. Robust covariance estimates were used. Group=2.00 was specified as the reference category. Wald chi-square statistics and corresponding p-values are reported.

Table S2. Multivariable Linear Regression Analysis of Depression, Anxiety, and Stress Scores Among Participants with Stroke

Outcome	R^2	Adjusted R^2	Age β (p)	NIHSS β (p)	Income β (p)
Depression	0.345	0.319	-0.171 (0.084)	0.550 (<0.001)	0.150 (0.136)
Anxiety	0.329	0.303	-0.219 (0.029)	0.505 (<0.001)	0.201 (0.049)
Stress	0.260	0.231	-0.248 (0.019)	0.466 (<0.001)	0.133 (0.211)

Models were adjusted for age, NIHSS score, and income–expense status. β represents standardized regression coefficients. R^2 = coefficient of determination; NIHSS = National Institutes of Health Stroke Scale.
